# Mechanical stimulation of human tendon stem/progenitor cells results in upregulation of matrix proteins, integrins and MMPs, and activation of p38 and ERK1/2 kinases

**DOI:** 10.1186/s12867-015-0036-6

**Published:** 2015-03-13

**Authors:** Cvetan Popov, Martina Burggraf, Ludwika Kreja, Anita Ignatius, Matthias Schieker, Denitsa Docheva

**Affiliations:** Department of Surgery, Experimental Surgery and Regenerative Medicine, Ludwig-Maximilians-University (LMU), Nussbaumstr. 20, D-80336 Munich, Germany; Institute of Orthopaedic Research and Biomechanics, University of Ulm, Helmholtzstr. 14, D-89081 Ulm, Germany

**Keywords:** Tendon stem/progenitor cells, Mechanical stimulation, Tendon-related genes, Collagen-binding integrins, Matrix metalloproteinases

## Abstract

**Background:**

Tendons are dense connective tissues subjected periodically to mechanical stress upon which complex responsive mechanisms are activated. These mechanisms affect not only the development of these tissues but also their healing. Despite of the acknowledged importance of the mechanical stress for tendon function and repair, the mechanotransduction mechanisms in tendon cells are still unclear and the elucidation of these mechanisms is a key goal in tendon research. Tendon stem/progenitor cells (TSPC) possess common adult stem cell characteristics, and are suggested to actively participate in tendon development, tissue homeostasis as well as repair. This makes them an important cell population for tendon repair, and also an interesting research target for various open questions in tendon cell biology. Therefore, in our study we focused on TSPC, subjected them to five different mechanical protocols, and investigated the gene expression changes by using semi-quantitative, quantitative PCR and western blotting technologies.

**Results:**

Among the 25 different genes analyzed, we can convincingly report that the tendon-related genes - fibromodulin, lumican and versican, the collagen I-binding integrins - α1, α2 and α11, the matrix metalloproteinases - MMP9, 13 and 14 were strongly upregulated in TSPC after 3 days of mechanical stimulation with 8% amplitude. Molecular signaling analyses of five key integrin downstream kinases suggested that mechanical stimuli are mediated through ERK1/2 and p38, which were significantly activated in 8% biaxial-loaded TSPC.

**Conclusions:**

Our results demonstrate the positive effect of 8% mechanical loading on the gene expression of matrix proteins, integrins and matrix metalloproteinases, and activation of integrin downstream kinases p38 and ERK1/2 in TSPC. Taken together, our study contributes to better understanding of mechanotransduction mechanisms in TPSC, which in long term, after further translational research between tendon cell biology and orthopedics, can be beneficial to the management of tendon repair.

**Electronic supplementary material:**

The online version of this article (doi:10.1186/s12867-015-0036-6) contains supplementary material, which is available to authorized users.

## Background

Tendons are able to transmit forces with minimal deformation or energy loss due to their unique hierarchically organized structure. The tendon extracellular matrix (ECM) is mainly composed of collagens (type 1, 3-6) and various proteoglycans, whilst the tendon cellular content is dominated by tenocytes. Within the tendon cell niche, Bi et al., [1] have reported the existence of a novel cell population possessing classical stem cell features such as self-renewing and multipotentiality. These cells were named tendon stem/progenitor cells (TSPC). TSPC are very closely related to the better known mesenchymal stem cells isolated from bone marrow, however they convey features distinguishable from other stem cells, namely the expression of tendon-related genes and the ability to form tendon-like tissue in vivo. Furthermore, it is suggested that TSPC are essential during tendon development and repair, and if their functions are disturbed, they can contribute to the progression of tendon pathologies [1]. Thus, TSPC represent a very important cell type for in-depth investigation of tendon cell behaviour, and their easy isolation and cultivation in vitro makes them useful and powerful tools for tendon researchers.

Within the tendon tissue, tendon cells interact with each other and with the proteins from the ECM [2]. These interactions are essential for the cells to sense and respond to mechanical loading, which in turn influences tendon metabolism [3]. The cells react to mechanical stimuli through complex mechanotransduction processes that can regulate the anabolic (ECM synthesis) and catabolic (matrix metalloproteinases expression and ECM degradation) pathways. In normal conditions, these processes are balanced and resulting in the maintenance of tendon homeostasis. However, changes in the equilibrium may lead to tendon pathology due to tissue degradation because of augmented ECM remodelling [4-6].

A major factor of the mechanotransduction process is the mechanical deformation of the ECM, which can affect cell actin cytoskeleton and thereby alter cell shape, motility and function. Mechanical forces can be transmitted by focal adhesion sites and cell-cell junctions [4,6]. The core components of focal adhesions are the integrin receptors; transmembrane heterodimers that can be activated by changes in the ECM or actin cytoskeleton and are mediating “outside-in” and “inside-out” signalling between the cell and the ECM [7]. Integrin signaling is initiated at the focal adhesion sites, which are membrane-associated platforms consisting of clustered, ECM-bound integrins as well as various enzymes, kinases, cytoskeletal and adaptor proteins (e.g. focal adhesion kinase, FAK, paxillin, p130cas) in the cytoplasm. Integrin adhesion triggers “outside-in” signaling which frequently synergizes with growth factor-dependent cascades and activates downstream proteins such as extracellular signal-regulated protein kinases 1 and 2 (ERK), p38 mitogen-activated protein kinases and c-Jun N-terminal kinases (Jnk) [2]. Furthermore, integrin-mediated anchorage and signaling can also regulate cell survival processes through the activation of protein kinase B (Akt) survival pathway [2,8].

So far, there are only few studies reporting on the phenotypic responses of tendon-derived cells to mechanical stress [5,9-12]. In particular, Fong et al., [10] and Mackley et al., [9] applied a microarray technology and studied the effects of mechanical load on the transcriptome of rat palmar flexor tendon cells and mouse embryonic tendon fibroblasts, respectively. These studies have suggested some candidate genes, such as transforming growth factors and cytoskeletal adaptor proteins, to be involved in the tendon cell mechanotransduction. However, the tendon mechanobiology is still not fully understood and it needs further scientific exploration. Here, we focussed on human TSPC from Achilles tendon and stimulated them with three different mechanical magnitudes for one or three days. As an experimental approach, we applied low cost analysis, by semi-quantitative and quantitative PCR, of 25 selected genes. To our knowledge, we can present for the first time novel data on the effect of mechanical stimulation on the expression of genes that are: 1) essential matrix components of the TSPC niche; 2) integrin receptors, which establish the necessary cell-matrix interactions and translate mechanical stimuli in signalling cascades; 3) matrix metalloproteinases, which are downstream targets of the integrin signalling, and in turn can remodel the TSPC niche; and 4) five key kinases from the integrin-mediated signalling pathways.

## Results

### Stem cell characteristics and tenogenic profile of TSPC

**Figure 1:**
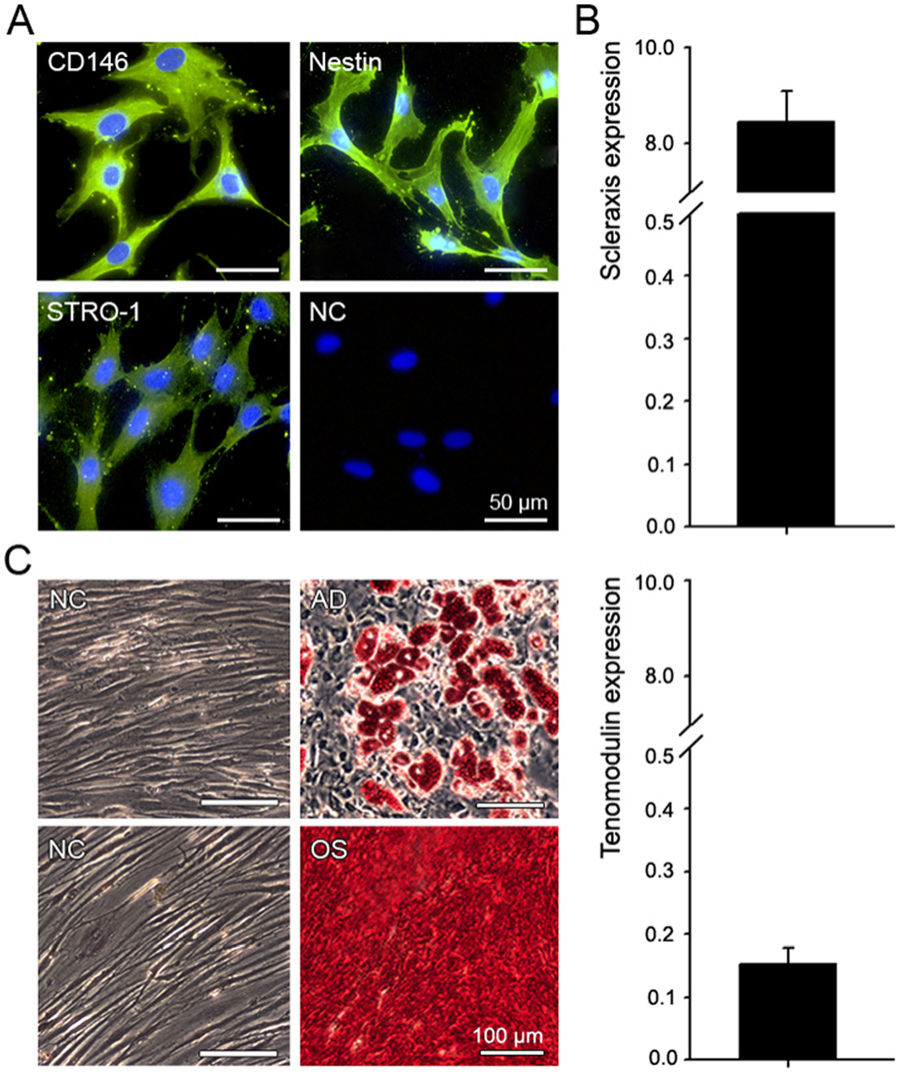
**Characterization of the human TSPC. (A)** Expression of CD146, Nesitn and STRO-1 stem cell markers demonstrated by immunocytochemistry; NC – negative control, cells incubated only with secondary antibody. Bar 50 μm. **(B)** Quantitative PCR analyses for Scleraxis and tenomodulin gene expression in TSPC, demonstrated as a ratio to HPRT housekeeping gene. **(C)** Adipogenic and osteogenic stimulation of TSPC for 21 days. Adipogenic (AD) and osteogenic (OS) differentiation visualized by Oil Red-O and Alizarin red staining, correspondingly; NC – negative control, unstimulated cells. Bar 100 μm. Data is representative of 3 donors, each used in 3 independent experiments.

**Figure 2:**
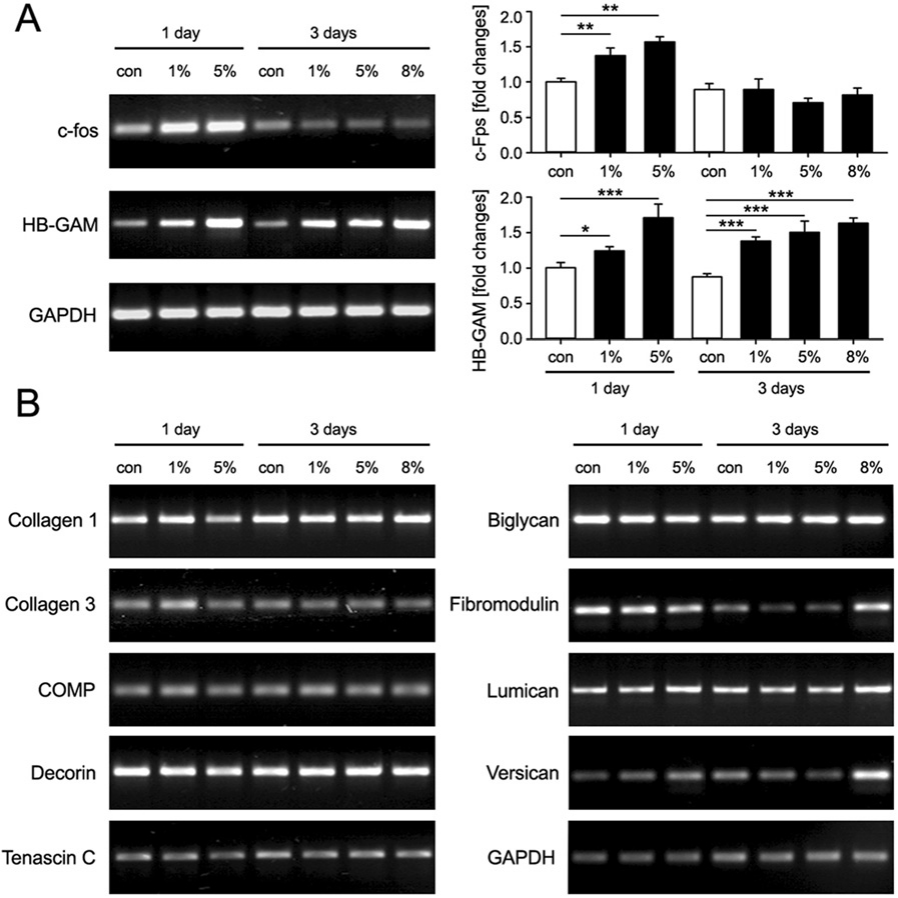
**Expression of mechanoresponsive and extracellular matrix genes upon mechanical stimulation of TSPC. (A)** RT-PCR for c-fos and HB-GAM expression changes after 1 and 3 days stimulation with 1, 5 and 8% mechanical stress. PCR densitometric quantification is shown as fold changes to the unstimulated controls; *p < 0.1, **p < 0.05, ***p < 0.01. **(B)** RT-PCR analysis for collagen 1 and 3, and tendon-related proteoglycans (COMP, decorin, tenascin C, biglycan, fibromodulin, lumican and versican). Data is representative of 3 donors, each used in 3 independent experiments.

TSPC were successfully isolated from human Achilles tendon biopsies [13] and their phenotype was re-validated in vitro based on the expression of stem cell surface markers (Figure 1A), tendon-related genes (Figures 1B and 2B) and the ability to undergo two-lineage differentiation (Figure 1C).

First, immunofluorescent staining for CD146, Nestin and STRO-1 stem cell markers demonstrated their ubiquitous expression in TSPC (Figure 1A). Next, quantitative PCR results confirmed the expression of the transcription factor Scleraxis and the tendon-specific gene tenomodulin in TSPC (Figure 1B). Additionally, the cells expressed collagen type 1 and 3, and several proteoglycans like cartilage oligomeric matrix protein (COMP), decorin, tenascin C, biglycan, fibromodulin, lumican and versican (Figure 2B), which are known to be highly expressed in tendons. Finally, we subjected TSPC to two different differentiation protocols, namely adipogenic and osteogenic stimulation (Figure 1C). Our results after 21 days of stimulation, demonstrated that TSPC could successfully commit towards adipocyte and osteoblast lineages visualized by Oil Red O (for lipid droplets) and Alizarin Red staining (for calcified matrix). Taken together, the above data reconfirmed the classical TSPC features.

### Mechanical loading of TSPC

In order to validate that TSCP have been subjected to mechanical stimulation, we analyzed the expression levels of well-known mechano-responsive genes such as c-fos and heparin-binding growth-associated molecule HB-GAM (Figure 2A). PCR analysis of c-fos demonstrated increased gene expression in TSPC stimulated mechanically for 1 day. Densitometric evaluation of the PCR bands showed that c-fos was significantly upregulated with 1.4 and 1.6 folds when stimulated with 1% or 5% loading strain, respectfully. At day 3, no effect of the stretching on c-fos expression was observed, as the gene expression levels remained similar to the non-stimulated cells. HB-GAM expression was clearly changed in response to the applied mechanical loading at day 1 and 3. The densitometric evaluation of the PCR bands demonstrated that at day 1, the HB-GAM expression was increased with 1.2 and 1.7 folds when stimulated with 1 and 5% strain, correspondingly. At day 3, a dose-dependent trend was observed and upon stimulation with 1, 5 and 8% strain, HB-GAM expression changed significantly with 1.4, 1.5 and 1.6 folds in comparison to the non-stimulated cells. Due to the observed expression changes in the tested mechano-regulated gene, we validated that TSCP were successfully stimulated with the applied axial stretching.

### Effect of mechanical loading on the expression of extracellular matrix genes

Next, we analyzed the effect of loading on the gene expression of ECM proteins that are highly expressed in the tendon tissue (Figure 2B). In particular, we investigated the expression of nine different genes - collagen type 1 and 3, COMP, tenascin C, decorin, biglycan, fibromodulin, lumican and versican. Our results showed that mRNA expression levels of the ECM genes have not been apparently altered by the mechanical stimulation and it mostly remained similar to that of non-stimulated controls at day 1 and day 3. Only in the case of fibromodulin, lumican and versican, we observed an increased expression at day 3 when TSPC were stimulated with 8% strain. These results suggested that 8% mechanical loading over a longer period have a positive effect on certain ECM genes expressed by the tendon cells. Therefore, we propose that in order to achieve a successful mechanical stimulation in TSPC, higher mechanical strain applied for extended time period is required.

### Analysis of integrin expression in response to mechanical stimulation

**Figure 3:**
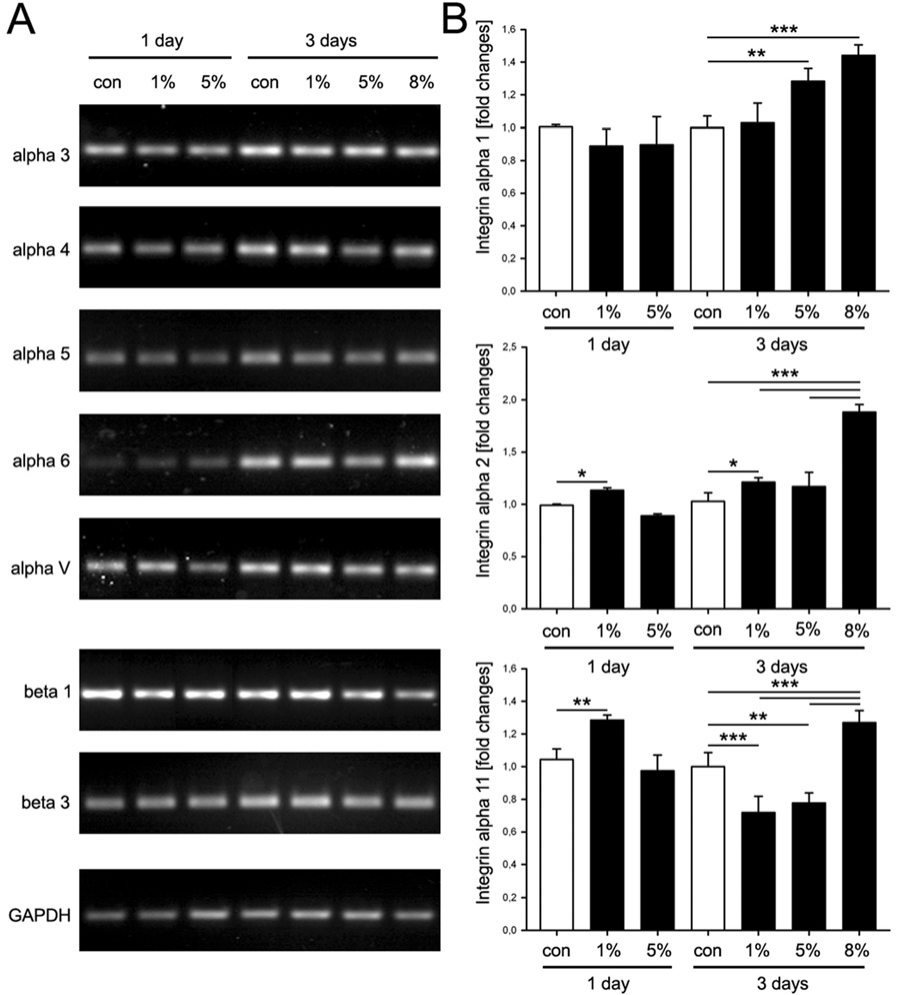
**Integrin expression changes after mechanical stimulation of TSPC. (A)** RT-PCR analysis of integrin alpha 3, 4, 5, 6 and V, and beta 1 and 3 in TSPC stimulated with 1, 5 and 8% strain for 1 and 3 days. **(B)** Quantitative PCR analysis for integrin alpha 1, 2 and 11 (fold change to the non-stimulated TSPC at day 1). Data is representative of 3 donors each used in 3 independent experiments; *p < 0.1, **p < 0.05, ***p < 0.01.

In order to sense and translate the applied external mechanical signals, cells implicate mechanoreceptors on their surface, such as the integrins. An individual integrin receptor consists of two non-covalently bound subunits – α and β [7]. In our study, we analyzed the expression changes of eight alpha (α1-6, α11 and αV) and two beta (β1 and β3) integrin subunits (Figure 3). RT-PCR analysis demonstrated a slight increase of integrin α3, α4, α5, α6 and αV subunit expression at day 3 in comparison to day 1 (Figure 3A). However, this difference was independent from the applied mechanical stress since it was also observed in the non-stimulated TSPC. Next, we analyzed the expression changes in the collagen-binding integrins α1, α2 and α11 by quantitative PCR (Figure 3B). We found that when stimulated for 3 days, TSPC upregulated integrin α1 with 1.3 and 1.5 folds upon 5 and 8% stress, correspondingly. The expression of integrin α2 was upregulated with 1.2 and 1.3 folds when stimulated with 1% strain at day 1 or day 3, respectively. The highest increase of integrin α2 (1.9 folds) was observed when TSPC were stimulated with 8% strain for 3 days. Mechanical loading of TSPC resulted in 1.3 folds increase in integrin α11 expression at day 1 when the cells were stimulated with 1% strain and at day 3, when stimulated with 8% strain. Regarding, integrin β-subunits, we did no observe any pronounced changes in between the control and mechanically stimulated TSPC. The expression levels of integrin β1 and β3 was similar also between the different days of stimulation. Taken together, our results suggest that 8% biaxial mechanical loading applied for 3 days modulated the expression levels of the three collagen I-binding alpha-subunits.

### Expression of matrix metalloproteinases upon mechanical stimulation

**Figure 4:**
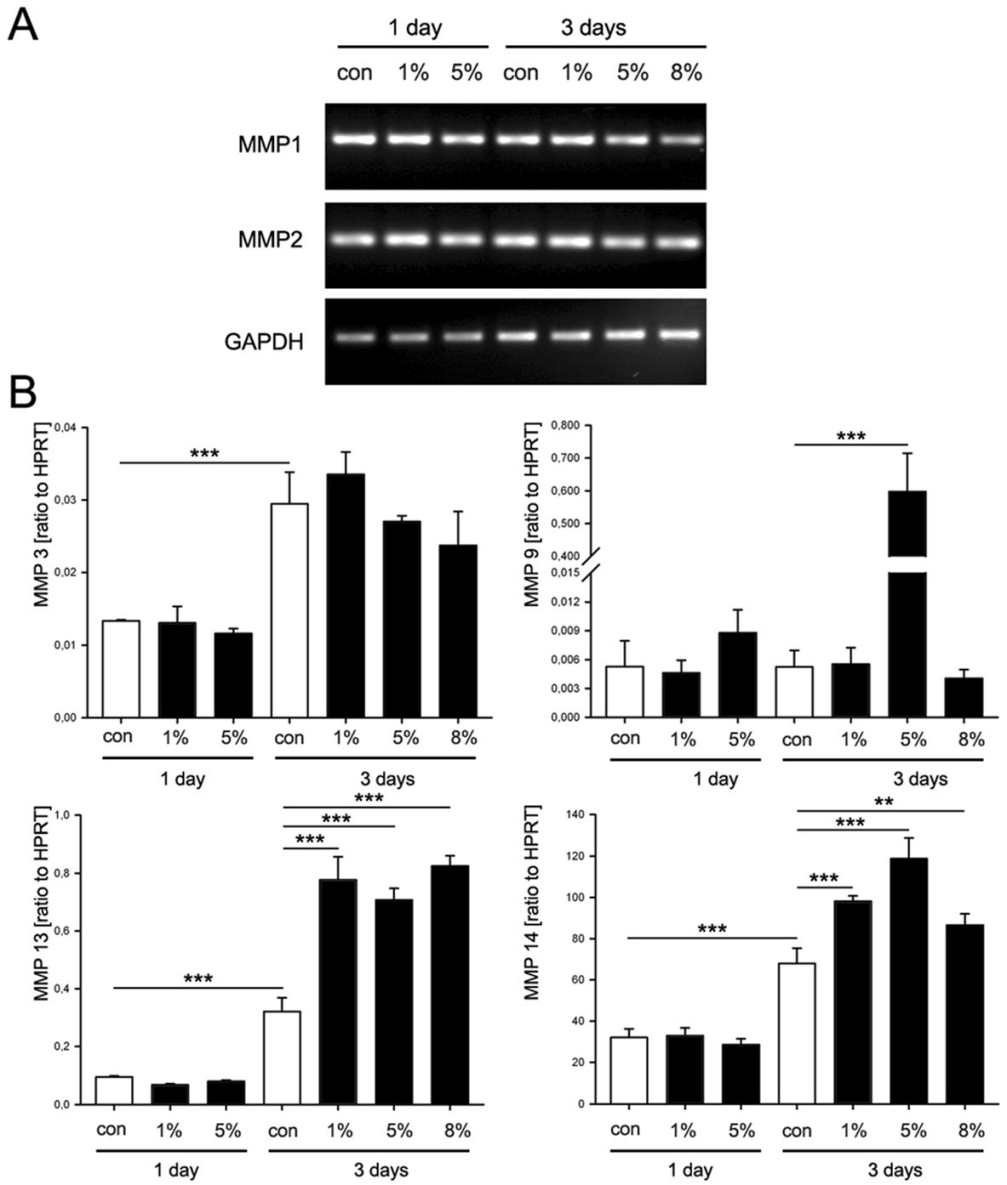
**Gene expression of matrix metalloproteinases upon mechanical stimulation of TSPC. (A)** RT-PCR analysis for MMP1 and MMP2. **(B)** Quantitative PCR analysis for MMP3, MMP9, MMP13 and MMP14 (ratio to HPRT housekeeping gene). Data is representative of 3 donors, each used in 3 independent experiments; *p < 0.1, **p < 0.05, ***p < 0.01.

Next, we examined the effect of mechanical loading on the gene expression changes of matrix metalloproteinases responsible for collagen degradation (MMP1, 2, 3, 9, 13 and 14). The expression of MMP1, 2 and 3 in TSPC did not respond to the mechanical stimulation as their gene levels remained similar to the non-stimulated cells at day 1 and 3 (Figure 4A and B). In contrast, the expression of MMP9, 13 and 14 increased when cells were stretched for 3 days (Figure 4B). The MMP9 expression was affected significantly by the 5% mechanical loading for 3 days as the gene levels increased with approximately 100 folds. When stimulated with 1, 5 or 8% loading strain, TSPC clearly upregulated MMP13 and 14, but only at day 3. In particular, MMP13 was increased with 2 folds at any of the applied loading strain. Similarly, MMP14 expression was elevated with at least 1.5 folds at day 3 in comparison to the non-stimulated TSPC. In conclusion, our data suggested that biaxial mechanical stimulation of TSPC for 3 days period of time upregulates the MMP9, 13 and 14 and is independently of the applied strain magnitude.

### Validation of gene expression changes on protein level upon 8% mechanical loading of TSPC

**Figure 5:**
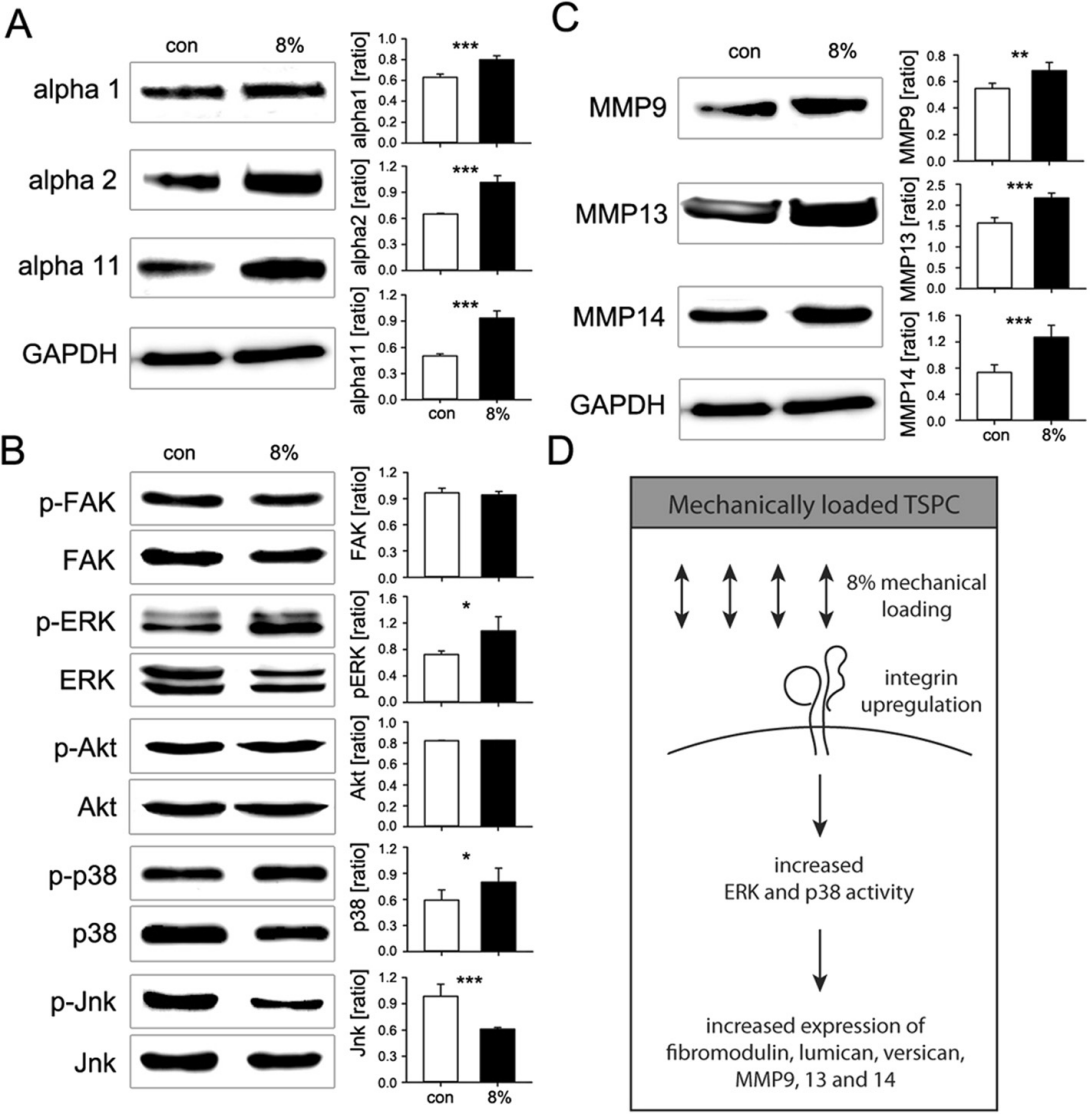
**Western blotting analysis for collagen-binding integrins, integrin-downstream kinases and matrix metalloproteinases upon 8% mechanical stimulation of TSPC. (A)** Collagen I-binding integrins α1, α2 and α11 (ratio to GAPDH protein expression); **(B)** Phosphorylated and total levels of FAK, ERK, Akt, p38 and Jnk (ratio phospho-/total protein); **(C)** MMP9, 13 and 14 (ratio to GAPDH). **(D)** Schematic summary of the changes occurring in TSPC upon 8% biaxial mechanical loading. Data is representative of 3 donors, each used in 3 independent experiments; *p < 0.1, **p < 0.05, ***p < 0.01.

Based on the obtained mRNA data, we performed western blotting analyses for the major candidate genes as we investigated: 1) the collagen binding integrins α1, α2 and α11; 2) the activation of five key integrin downstream kinases and 3) the expression of MMP9, 13 and 14 responsible for the collagen degradation. For this analysis, TSPC were stimulated with 8% biaxial loading for 3 days, since this was the best condition according to our mRNA screening. Similar to the mRNA results (Figure 3B), stimulation with 8% mechanical strain resulted in significantly higher protein production for all three integrins. However, the increase in the protein expression was more pronounced for integrin α2 (1.5 folds) and α11 (1.9 folds) (Figure 4A). Next, we analyzed the changes in the activity of five integrin downstream kinases FAK, ERK, Akt, p38 and Jnk (Figure 5B). We found that upon mechanical loading, the activity of ERK (1.5 folds) and p38 (1.4 folds) kinases significantly increased, whereas the activity of Jnk (1.6 folds) was significantly reduced in the mechanically loaded TSPC. The other two kinases – FAK and Akt were not influenced by the mechanical loading. The analysis of MMP protein levels (Figure 5C) demonstrated that upon mechanical stimulation the expression of MMP9, 13 and 14 were significantly increased as the most pronounced changes were observed in the expression of MMP13 (1.4 folds) and 14 (1.8 folds) in comparison to the non-stimulated TSPC.

## Discussion

Despite of the acknowledged importance of mechanical loading for tendon development and healing, the effect of different loading magnitudes on tendon cells has not been studied in details. Thus, with our study we shade a light on the changes in the gene expression of tendon-related matrix proteins, integrins and MMPs occurring after different mechanical loadings – 1, 5 and 8%, applied for one or three days. As a cell source, we used TSPC isolated from human Achilles tendon [13], because they are not only an important cell type in tendon development and repair, but also a potential clinical cell source for tendon regeneration. A limitation of the present study was the use of only three TSPC biological replicates due to the difficult obtainability of Achilles tendon biopsies from young and healthy patient donors. Thus, in future studies an increased cohort of TSPC donors has to be investigated.

In previous publication, we have already reported the isolation and initial characterization of TSPC [13]. These cells express common stem cell surface markers such as CD146, Nestin and STRO-1, which are characteristic for stem cells from different tissue sources [14,15]. For example, CD146 was shown as marker of stem cells localized in the vascular wall [16], Nestin was found predominantly in multipotent cells from the central nervous system [17] and STRO-1 was expressed on mesenchymal stem cells in bone marrow [18]. Bi et al., [1] and Tempfer et al., [19] have also determined the expression of these three markers in TSPC. Additionally to the expression of stem cell-related markers, TSPC preserve the expression of the typical tendon lineage genes namely Scleraxis [20] and tenomodulin [21]. Here, we have reconfirmed the TSPC characteristics by: 1) the expression of CD146, Nestin and STRO-1; 2) the expression of Scleraxis and tenomodulin; and 3) the ability of these cells to commit to adipogenic and osteogenic lineages. Hence, we concluded that TSPC represent a good model system to study the behavior of the tendon-derived cells under mechanical stress in vitro.

In vivo, the whole tendon unit can be subjected to tissue stretching that normally does not exert 4%. This stretching is considered as a maximum of the physiological range. In some rare occasions, the applied forces can surpass that range. Then, this can result in formation of microscopic tears in the collagen fibers. Extreme cases, when the generated forces exerted 8-10% tissue stretching or prolonged sub-physiological loadings can cause tissue rupture and thereon tendon failure [6]. However, studies have shown that avian flexor and rabbit Achilles tendons can withstand stretching up to 14% and 16%, respectively [6,22] suggesting that the tendon strain might be under-estimated. With regards to the cells within the tendon tissue, it has been proposed that up to 10% mechanical stretching of tendon fibroblast is within their physiological range [22]. The cell response to mechanical stimuli depends mainly on the type of loading (static or cyclic, uniaxial or biaxial), stretching magnitude, frequency and duration [22]. Several studies, performed with tendon fibroblasts obtained from various species have used mechanical stimulation (cyclic uniaxial or biaxial) in the range of 4 to 10% stretching [22]. Therefore, we stimulated TPSC from three different biological replicates with three magnitudes located in the low (1%), middle (5%) and high (8%) region of the physiological range. We applied the mechanical stress for two different time periods, one and three days, in order to study short and long-term effects. To confirm the successful mechanical loading of TSPC, we first investigated the expression of short-term and long-term mechanically stimulated genes c-fos and HB-GAM. The transcription factor c-fos is established short-term mechanically-regulated gene, which peaks after 30 min upon mechanical stress [23]. HB-GAM gene was upregulated in hMSC upon cyclic mechanical stimulation as the gene expression was detected after longer periods of mechanical loading (48 hours) [24]. Our results clearly demonstrated that upon mechanical stimulation the expression levels of both mechano-regulated gene markers c-fos and HB-GAM were increased, as in the case of HB-GAM the increased gene expression was detected in each of the two different time points and it was in correlation to the strength of the biaxial mechanical load.

Then, we analyzed the changes in the expression levels of collagen 1 and 3 genes, which are key matrix proteins in the TSPC niche. Few articles have suggested that mechanical stimulation has a positive effect on the collagen 1 expression. For example, in human tendon fibroblasts from anterior cruciate ligament, 4% and 8-10% cyclic uniaxial stimulation resulted in the induction of collagen 1 gene expression [25]. Howard et al., [26] reported an increase of collagen 1 and fibronectin expression in human ligament fibroblasts when stimulated with 5% cyclic biaxial stretch, but observed no effect on collagen 1 expression by 10% stretching [22]. Interestingly, Hsieh et al., [27] suggested that different tendon types can be influenced differently by the mechanical loading. In human anterior cruciate ligaments, 7.5% biaxial stretch increased collagen 1 and decreased collagen 3 expression. In contrast, in medial collateral ligaments the same mechanical loading led only to elevation of the collagen 3 expression, while collagen 1 expression remain unchanged [22]. In TSPC, our PCR results demonstrated no changes in the expression levels of collagen 1 and 3 at the three different magnitudes and at the two different time points. Hence, we can conclude that TSPC isolated from human Achilles tendon do not elevate the mRNA levels of collagen 1 and 3 when mechanically stimulated. Taking into account the complex post-translational modification of collagens, it remains to be further clarified if mechanical stress might influence the protein levels of collagen 1 and 3 in TSPC.

Next, we studied the expression levels of proteoglycans that are characteristic for the tendon tissue. We detected an increased expression of fibromodulin, lumican and versican when TSPC were stimulated with 8% axial loading, while the levels of COMP, decorin, tenascin C and biglycan remained unchanged. The observed increase of fibromodulin, lumican and versican RNA levels upon mechanical loading can be explained by the function of these proteoglycans in the tendon tissue. Fibromodulin and lumican were found to be important in the fibrilogenesis and maturation of the collagen fibers [28], while versican is involved in cell adhesion, proliferation and migration as well as in ECM assembly [29]. Little is known about the alterations in proteoglycan expression levels in tendons, triggered by mechanical loading. Only few studies have reported that decorin and biglycan expression was not influenced by mechanical stimulation [5,30,31], while versican was significantly upregulated [31]. Taken together, our results are in line with the above literature and demonstrated that high levels of mechanical stress can upregulate the gene expression of fibromodulin, lumican and versican. It will be of great interest to further explore how these proteoglycans affect the TSPC functions upon mechanical stress, which can be the focus of follow up studies.

Integrins can convert the mechanical signals into cytoplasmic signals via outside-in signaling cascade, which affects a number of cellular processes including cell proliferation, migration and differentiation, gene expression, matrix remodeling, cytoskeletal dynamics and cell survival [3,7]. Subramony et al., [32] have reported that mechanical stimulation of hMSC can trigger the upregulation of integrin α2, α5 and β1 after 7 or 14 days of 1% axial stimulation. The authors concluded that the increase in integrin expression is important to ensure the sufficient numbers of surface receptors that are necessary to sense and translate the mechanical stimuli in signaling cascade in MSC [32]. With regards to TSPC, to our knowledge, this study delivers the first data on the effect of mechanical stimulation on the RNA expression of different integrins responsible for collagen (α1, α2 and α11), fibronectin (α3, α4, α5 and αV) and laminin (α6) binding. The RT-PCR analysis performed for integrin α3, α4, α5, α6 and αV demonstrated increased integrin expression at day 3. However, that expression did not dependent on the magnitude of the mechanical stimulation. In contrast, the expression of collagen-binding integrins were strongly upregulated at day 3 on mRNA and protein levels when TSPC were stimulated with 8% mechanical stress, suggesting that this mechanical loading was optimal to trigger molecular response in TSPC. Taken together, we are the first to report integrin expression changes occurring in TSPC upon mechanical stimulation.

Based on these initial findings, we investigated further the integrin-orchestrated signaling cascade in TPSC triggered by the mechanical stimulation. Involvement of several different kinase pathways from the signaling cascade of integrin-transmitted mechanical stimuli was already demonstrated in cell, delivered from muscles [33,34], cartilage [35-37] and bone [38,39] tissues. A common tendency between all musculoskeletal tissues was the activation of ERK signaling pathway upon mechanical loading. With regards to other kinases, the published data is contradictive. For example, Zhou et al., [35] and De Croos et al., [36] found that mechanical stress in human and bovine chondrocytes results in increased Jnk phosphorylation, whereas Xu et al., [37] did not confirmed this observation in rat chondrocytes, in which no changes in the phospho-Jnk levels were detected. Others have demonstrated that integrin-dependent transmission of the mechanical signaling in the osteoblasts and chondrocytes was mediated through increased FAK [38], p38, Jnk and Akt activity [40-42]. Matsui et al.,[41] suggested that the activation of the different kinases might correlate to the magnitude of the mechanical stress: when MC3T3 cells were treated with lower cyclic mechanical loading ERK phosphorylation was elevated, whereas when MC3T3 were subjected to higher mechanical stretch Jnk and p38 active forms were also increased. Our protein results from TSPC treated with 8% mechanical loading clearly demonstrated a significant increase in ERK and p38 phosphorylation. This result suggests that 8% biaxial mechanical loading is an optimal magnitude of mechanical loading for TSPC. In contrast to MC3T3, TSPC stimulated with 8% mechanical loading had decreased Jnk activity, a finding that needs to be further investigated.

Finally, we analyzed the expression of the matrix metalloproteinases. It is known that binding of the integrins to their ECM ligands results in increased expression of various MMPs, which participate in the important cell processes such as cell migration as well as matrix remodeling [43]. Increased MMP expression upon mechanical loading without or with presence of other factors has been previously demonstrated in tenocytes [44,45], chondrocytes [46] and osteoblasts [47]. This effect, however, depends strongly on the cell type, the duration and the magnitude of the applied stress. For example, Archambault J et al. [48] found an increased MMP-1 and MMP-3 expression in rabbit Achilles tendon cells when stimulated with IL-1β and 5% cyclic biaxial stretching. Oppositely, Sun et al. [49] reported that in human synovial cells that were stimulated with 2% cyclic stretch, the expression levels of MMP-1 and MMP-13 decreased. Our analysis clearly demonstrated that 8% biaxial mechanical stress of TSCP for 3 days significantly increased MMP9, 13 and 14 expressions in comparison to the non- and to 1 day stimulated cells.

## Conclusions

To our knowledge, our study is the first one to address the effect of different mechanical loadings on the expression of selected genes in human TSPC isolated from Achilles tendon. Here, we showed that biaxial mechanical stress induces the expression of the proteoglycans fibromodulin, lumican and versican; collagen-binding integrin receptors α1, α2 and α11; and MMP9, 13 and 14 via ERK and p38 kinase activation (Figure 5D). These proteins play an important role for TSPC’s niche composition, cell survival, mechanosignaling and matrix remodeling. Furthermore, we established an efficient experimental protocol for the mechanical stimulation of human TSPC and we can propose 8% mechanical strain for 3 days as an optimal setup that promotes mechanoresponse and gene expression changes in these cells. Taken together, we believe that our study contributes to a better understanding of the TSPC and their response to mechanical stimuli, and it can serve as an experimental model for further in-depth analysis of the mechanotransduction mechanisms in tendon cells.

## Methods

### Cell isolation and cultivation

Achilles tendon biopsies derived from three young and healthy human patients (male, age 28 ± 5 years), who had undergone surgical operations due to lower extremity accidents in the Surgical Clinic of Ludwig-Maximilians-University in Munich (LMU). TSPC were isolated and initially characterized by Kohler et al., [13]. The procedure was approved by the Ethical Commission of the LMU Medical Faculty (grant No. 166-08) and written informed consent was obtained from all donors. Briefly, TSPC were isolated as tendon tissue (without the paratenon) was minced into small pieces and enzymatically digested overnight with 0.15% collagenase II (Worthington, USA). Then the digested tissue was filtered (100 μm nylon mesh) and centrifuged for 10 min. The cell pellet was suspended in DMEM/Ham’s F-12 supplemented with stabile glutamine, 1% MEM-Amino-acids (Biochrom, Germany), 10% FBS and 1% L-ascorbit-acid-2-phosphate (Sigma-Aldrich, Germany) [13]. The cell suspension was plated on polystyren dishes and the obtained TSPC were expanded in a humidified incubator at constant 37°C and 5% CO2 and then used in different experiments at passages 4-6 or stored in liquid nitrogen tank.

### Immunocytochemistry

TSPC at passage 6 were plated and cultured on 20 μg/ml collagen 1-coated glass slides (BD Bioscience, USA) for 48 h. Then, the cells were fixed with 4% paraformaldehyde (Merck, Germany), permeabilized with Triton X100 (Sigma-Aldrich) and blocked with 3% BSA (PAA, USA). Primary antibodies against CD146 (Millipore, USA), Nestin (Proteintech, USA) and STRO-1 (R&D Systems, USA) were applied overnight at 4°C. Next, secondary Alexa Flour 488-conjugated antibodies and DAPI were used (all Life technologies, USA). As negative control were used cell-seeded slides which were incubated only with secondary Alexa Flour 488-conjugated antibodies and DAPI. Photomicrographs were taken with Axiocam MRm camera on Axioskope 2 microscope (Carl Zeiss, Germany). Staining procedures were reproduced at least twice.

### Cell differentiation

TSPC were stimulated at passage 5 towards adipogenic and osteogenic lineages. For adipogenic differentiation, TSPC were plated in 6-well dishes (1 x 10^5^ cells/cm^2^). Cells were stimulated for 21 days using the induction medium composed of DMEM-high glucose medium, 10% FBS, 1 μM dexamethasone, 0.2 mM indomethacin, 0.1 mg/ml insulin and 1 mM IBMX (all Sigma-Aldrich). The extent of adipogenic differentiation was evaluated by standard Oil Red-O staining.

For osteogenic differentiation, 3.5 x 10^4^ cells/cm^2^ were seeded in 6-well dishes and cultivated in osteogenic medium composed of DMEM high glucose, 10% FBS, 10 mM β-glycerophosphate, 50 μM L-ascorbic-acid-2-phosphate and 100 nM dexamethasone (all Sigma). After 21 days, Alizarin Red staining was performed using the Osteogenic Quantification kit (Millipore). After each differentiation experiment, photomicrographs were taken with AxiocamICc3 camera mounted on AxiovertS100 microscope (Carl Zeiss).

### Mechanical stimulation

Biaxial mechanical stimulation of the TSPC from three different donors at passage 4-6 was performed in a six-station stimulation apparatus driven by eccentric motor [50]. For this, 1 × 10^5^ cells were plated and cultured for 4 days on FBS-coated flexible silicone dishes (60 mm × 30 mm). Then, triplicate dishes were stretched cyclically in the long axis at a frequency of 1Hz and a magnitude of 1%, 5% or 8% (corresponding to 1 × 10^4^, 5 × 10^4^ or 8 × 10^4^ μstrain) continuous for 60 min (1 day) or intermittent on 3 consecutive days for 60 min/day (3 days). In parallel, non-stimulated cells, plated on flexible silicone dishes, were used as controls on day 1 and day 3, respectively. Directly after stimulation, cells were lysed and subjected to mRNA isolation.

### Reverse transcriptase and quantitative PCR analysis

Total RNA was extracted directly after stimulation with RNeasy Mini Kit (Qiagen, Germany). Then, total RNA concentration was determined by Nanodrop (Thermo scientific, USA) and its integrity was verified by 28S/18S rRNA ratio on agarose gel electrophoresis (Peqlab, Germany). For cDNA synthesis, 1 μg total RNA and AMV First-Strand cDNA Synthesis Kit (Life technologies) were used. RT-PCR was performed with Taq DNA Polymerase (Life technologies) in MGResearch instrument (BioRad, Germany). Primer pairs and PCR conditions are listed in Additional file 1: Table S1. For c-Fos and HB-GAM, PCR bands were quantified densitometrically using the BioCapt software (Vilber Lourmat, Germany). Values were normalized to GAPDH and results reported as fold change to the none-stimulated cells.

For quantitative PCR, LightCycler Fast Start DNA Master SYBR Green kit (Roche, Germany) and target-specific, company designed and validated primer kits for Scleraxis, tenomodulin, α1, α2, α11, MMP3, MMP9, MMP13, MMP14, HPRT and GAPDH (Search-LC, Germany) were used. The quantitative PCR was performed in LightCycler1.5 instrument (Roche) equipped with LightCycler 3.5.3 software. Crossing points for each sample and inter-run calibrator were determined by the second derivative maximum method and relative quantification was performed using the comparative ΔΔCt method with efficiency correction according to the manufacturer’s protocol. The relative gene expression was calculated as a ratio to GAPDH or HPRT, depending on the expression levels (high or low) of the targeted genes. GAPDH and HPRT were selected for normalization due to reported stability upon mechanical loading [51,52]. Detailed information about qPCR template and conditions can be found in Additional file 2: Table S2. All PCR results have been reproduced three independent times.

### Western blot analysis

Total protein from TSPC (none-stimulated or with 8% mechanical loading) at passage 6 was isolated according to Alberton et al., [53]. In brief, adherent cells were lysed in 1x Cell Culture Lysis Reagent (Promega, 25 mM Tris-phosphate pH 7.8, 2, mM DTT, 2 mM 1,2-diaminocyclohexane-N,N,N’,N’-tetraacetic acid, 10% glycerol, 1% Triton X-100). Total protein was quantified with Micro BCA protein assay kit (Pierce, USA). Aliquots of 20 μg were denatured at 99°C for 5 min and loaded on SDS-PAGE gels. Then, proteins were transferred onto PVDF membrane, blocked with 5% skim milk (Merck) and incubated in primary anti-human antibodies: integrins α2 (BD Bioscience) and α11 (R&D Systems), phospho-FAK (Life technologies), total FAK, total and phospho-ERK1/2; p38, Jnk and MMP9 (all Cell Signaling, USA), MMP13 and MMP14 (Thermo scientific, USA) and GAPDH loading control (Merck) overnight at 4°C. Secondary HRP-conjugated antibodies (Rockland, USA or Cell Signaling) were applied for 1 h at room temperature. Western blots were visualized with ECL solution (GE Healthcare, USA) as photomicrographs were taken on ImageQuant LAS 4000 mini (GE healthcare, USA) as bent size was quantify by using the machine software.

### Statistics

Statistical evaluation was performed using the GraphPad Prism 5 software (GraphPad Software, USA). N = 3 means that the results of three different TSPC donors were pull together as for each donor we used a mean value of three independent experiments (N = 3). Graphs and bar charts show mean values and standard deviation. Unpaired *t*-test was used for each condition versus the control (non-stimulated TSPC). A p-value <0.05 was considered statistically significant (*p < 0.05; **p < 0.01, ***p < 0.001).

## Additional files

Additional file 1:
**PCR primers and conditions.**
Additional file 2:
**qPCR checklist according to MIQE.**

## Abbreviations

TSPC: Tendon stem/progenitor cells
MMP: Matrix metalloproteinases
ERK1/2: Extracellular-signal-regulated kinases 1 and 2
p38: p38 Mitogen-activated protein kinases
ECM: Extracellular matrix
JNK: c-Jun N-terminal kinases
Akt: protein kinase B
COMP: Cartilage oligomeric matrix protein
FAK: Focal adhesion kinase
hMSC: Human mesenchymal stem cells
MSC: Mesenchymal stem cells
IBMX: 3-Isobutyl-1-methylxanthine and FBS, fetal bovine serum
