# Supplementary material for: Mechanical stimulation of human tendon stem/progenitor cells results in upregulation of matrix proteins, integrins and MMPs, and activation of p38 and ERK1/2 kinases
Source: BMC Mol Biol. 2015 Mar 13;16:6. doi: 10.1186/s12867-015-0036-6 (PMC4373449; doi:10.1186/s12867-015-0036-6)
Supplement: Additional file 2: — qPCR checklist according to MIQE. [file 12867_2015_36_MOESM2_ESM.docx]

| **Target gene**  **(Acc. number)** | **Primers** | **Ann. T, °C** | **Size,**  **bp** | **Reference** |
| --- | --- | --- | --- | --- |
| **c-fos**  (NM_005252) | F 5´-ctccggtggtcacctgtact-3´  R 5´-ggtgaagacgaaggaagacg-3´ | 50°C | 59 | Self designed |
| **HB-GAM**  (NM_002825) | F 5´-gcaaaccatgaagacccaga-3´  R 5´-ggcttggagatggtgacagt-3´ | 55°C | 189 | Seefried L, 2010, Eur Cell Mater |
| **Collagen I**  (NM_000088) | F 5´-catctccccttcgtttttga-3´  R 5´-ctgtggaggagggtttcaga-3´ | 54°C | 594 | Lin Y, 2005, J Cell Mol Med |
| **Collagen III**  (NM_000090) | F 5´-ttataaaccaaactctatct-3´  R 5´-tattatagcaccattgagac-3´ | 42°C | 260 | Boykiw R, 1998, Matrix Biol |
| **COMP**  (NM_000095) | F 5´-gctctgtggcatacaggaga-3´  R 5´-catagaatcgcaccctgatg-3´ | 53°C | 145 | Tian H, 2006, Acta Biochim Biophys Sin |
| **Decorin**  (NM_001920) | F 5´-gctggaccgtttcaacagagag-3´  R 5´-tcattctcatgggcacgcag-3´ | 54°C | 437 | Chung EJ, 2002, Mol Cells |
| **Tenascin C**  (NM_002160) | F 5´-gagaaaggcagacacaagag-3´  R 5´-gcagtccagttgagtttgag-3´ | 57°C | 395 | Self designed |
| **Biglycan**  (NM_001711) | F 5´-gatggcctgaagctcaa-3´  R 5´-ggttgttgaagaggctg-3´ | 52°C | 406 | Boykiw R, 1998, Matrix Biol |
| **Fibromodulin**  (NM_002023) | F 5´-ctggaccacaacaacctgac-3´  R 5´-ggatcttctgcagctggttg-3´ | 55°C | 442 |  |
| **Lumican**  (NM_002345) | F 5´-ctgcagtggctcattcta-3´  R 5´-gacctccaggtaatagtt-3´ | 46°C | 576 |  |
| **Versican**  (NM_004385) | F 5´-gatgtgtattgttatgtgga-3´  R 5´-catcaaatctgctatcaggg-3´ | 51°C | 310 |  |
| **Integrin α3**  (NM_002204) | F 5´-atcttgagagccacagtca-3´  R 5´-ctgggtccttctttctagttc-3´ | 52°C | 201 | Lin KT, 2005, J Cell Mol Med |
| **Integrin α4**  (NM_000885) | F 5´-aatggatgagacttcagcact-3´  R 5´-ctcttctgttttcttcttgtagg-3´ | 48°C | 278 |  |
| **Integrin α5**  (NM_002205) | F 5´-actaggaaatccattcacagttc-3´  R 5´-gcatagttagtgttctttgttgg-3´ | 52 °C | 201 |  |
| **Integrin α6**  (NM_000210) | F 5´-cttggagaagatgggtttatt-3´  R 5´-gaatacagataggggaggaaa-3´ | 48°C | 213 |  |
| **Integrin αv**  (NM_002210) | F 5´-ggagcacatttagttgaggtat -3´  R 5´-actgttgctaggtggtaaaact-3´ | 46°C | 274 |  |
| **Integrin β3**  (NM_000212) | F 5´-ctgctgtagacatttgctatga-3´  R 5´-gccaagaggtagaaggtaaata-3´ | 52 °C | 211 |  |
| **Integrin β5**  (NM_002213) | F 5´-ctgtggactgatgtttcctt-3´  R 5´-gtatgctggttttacagactcc-3´ | 54°C | 407 |  |
| **MMP1**  (NM_002421) | F 5´-cctagctacaccttcagtgg-3´  R 5´-gcccagtacttattcccttt-3´ | 50°C | 338 | Kim S, 2008, Exp Dermatol |
| **MMP2**  (NM_001127891) | F 5´-gctcagatccgtggtgagat-3´  R 5´-ggtgctggctgagtagatcc-3´ | 54°C | 207 | Satpathy M, 2009, J Biol Chem |
| **GAPDH**  (BC029618) | F 5´-caactacatggtttacatgttc-3´  R 5´-gccagtggactccacgac-3´ | 50°C | 181 | Docheva D, 2010, Eur Cell Mater. |
